# Microphysiological stem cell models of the human heart

**DOI:** 10.1016/j.mtbio.2022.100259

**Published:** 2022-04-14

**Authors:** Ulgu Arslan, Alessia Moruzzi, Joanna Nowacka, Christine L. Mummery, Dominik Eckardt, Peter Loskill, Valeria V. Orlova

**Affiliations:** a Department of Anatomy and Embryology, Leiden University Medical Centre, Leiden, the Netherlands; b NMI Natural and Medical Sciences Institute at the University of Tübingen, Reutlingen, Germany; c Institute for Biomedical Engineering, Eberhard Karls University Tübingen, Tübingen, Germany; d Miltenyi Biotec B.V. & Co. KG, Bergisch Gladbach, Germany; e 3R-Center for *in Vitro* Models and Alternatives to Animal Testing, Tübingen, Germany

**Keywords:** Human induced pluripotent stem cells, Cardiac microtissue, Engineered heart tissue, Heart-on-chip, Cardiomyocyte maturation, Multicellular cell diseases and drug efficacy platform, Structural readout, Functional readout

## Abstract

Models of heart disease and drug responses are increasingly based on human pluripotent stem cells (hPSCs) since their ability to capture human heart (dys-)function is often better than animal models. Simple monolayer cultures of hPSC-derived cardiomyocytes, however, have shortcomings. Some of these can be overcome using more complex, multi cell-type models in 3D. Here we review modalities that address this, describe efforts to tailor readouts and sensors for monitoring tissue- and cell physiology (exogenously and *in situ)* and discuss perspectives for implementation in industry and academia.

## Graphical abstract

**Figure:**
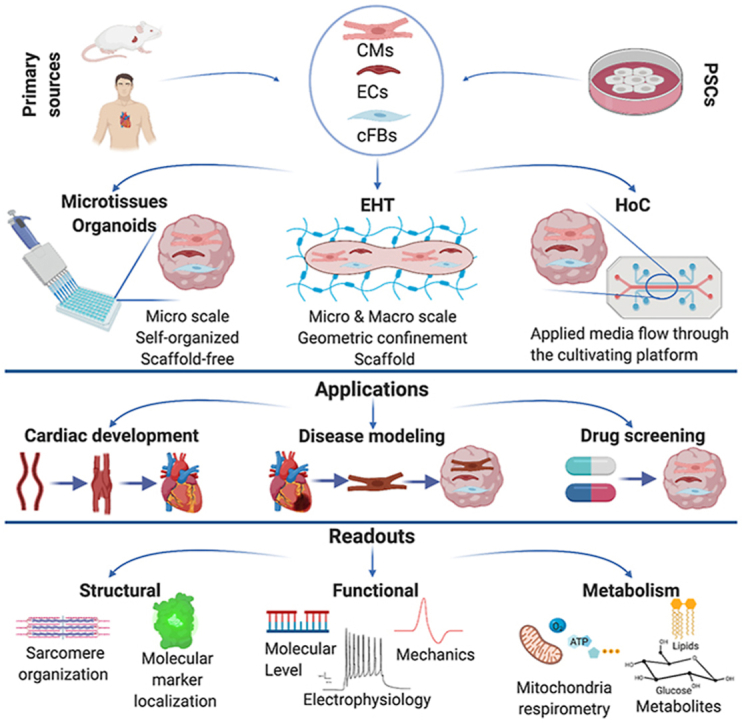

## 1 Introduction

Animal models have been very useful in understanding how the heart develops and the molecular mechanisms underlying congenital heart defects. They have been less successful in identifying the genetic and other causes of cardiac dysfunction or predicting the effects of drugs, largely because of differences in animal and human physiology. The advent of human pluripotent stem cells (hPSCs) [1,2] has however opened new avenues to study the human heart and its pathologies. We review here state-of-the-art models now being used to investigate the human heart, how they are being used to monitor human heart (patho-)physiology, their shortcomings and the expectations for the future.

Cardiomyocytes (CM) were among the first cell types to be differentiated from hPSCs [3,4], specifically from human embryonic stem cells (hESCs). Differentiation protocols initially entailed culture of undifferentiated cells as aggregates in suspension, called “embryoid bodies”, in the presence of growth factors. Since these first reports, many groups have developed methods to differentiate CMs in 2D (reviewed in Ref. [5]) and to form three dimensional (3D) cardiac tissues (Table 1). Whilst strikingly evident because of their cyclic contraction, at most 5% of the differentiated cells were initially CMs. This meant that most analysis was based on methods suitable for single cells such as patch clamp electrophysiology and immunostaining of sarcomeric proteins. hPSC-derived CMs (hPSC-CMs) were also immature compared to primary adult CMs with respect to their morphology, contractility, electrophysiology, metabolism and gene expression [[6], [7], [8]]. Nevertheless, various studies have shown that hPSC-CMs can be valuable for disease modelling and drug screening even if the CMs are immature, especially if they are derived from human induced pluripotent stem cells (hiPSCs) from patients [[9], [10], [11], [12], [13]]. Notably, all cardiac ion channels are expressed in hPSC-CMs so that modelling channelopathies caused by mutations in ion channel genes has been particularly successful. hiPSCs carry the genetic background of the individuals from whom they are derived and thus allow diseased genomes or disease predisposition to be captured in the derivative cardiac cell types; they thus support more personalized approaches to disease modelling and drug screening.

**Table 1:** Literature summary of microtissues/organoids.

Study	Cell types	Application	Readouts	
			Structural	Functional
Microtissues/Organoids				
[80] Archer et al., 2018	hiPSC-CM Primary human cardiac ECs Primary human cFBs	Drug Screening	Histology Confocal Imaging – immunofluorescence	Gene expression (qRT-PCR) Cell viability assay (cellular ATP content)
[14,17] Beauchamp et al., 2015; 2020	hiPSC-CM human embryonic-cFBs	Cardiac development Disease modelling -cardiac fibrosis	Confocal imaging-immunofluorescence Electron microscopy Histology	Cell viability assay (live-dead staining) Ca^2+^ imaging Pharmacological analysis Gene expression (qRT-PCR) Western Blot Motion analysis Patch clamp
[28] Drakhlis et al., 2021	hECS-CM	Cardiac development	Histology Multiphoton microscopy –immunofluorescence Live Optical fluorescence imaging - reporter cells; DiI-Ac-LDL Cell composition analysis – Flow cytometry Electron microscopy	Ca^2+^ imaging Patch clamp Gene expression (microarray, RNAseq)
[22] Giacomelli et al., 2017	hiPSC or hECS-CM hiPSC or hESC-cardiac EC	Cardiac development Cardiac Maturation	Optical Microscopy Confocal Imaging-immunofluorescence	Gene expression (qRT-PCR) FACS Motion analysis Patch clamp MEA analysis
[18] Giacomelli et al., 2020	hiPSC-CM hiPSC-cardiac EC hiPSC-cFBs	Cardiac development Cardiac maturation Disease modelling -arrtyhmogenic cardiomyopathy	Confocal Imaging-immunofluorescence Electron microscopy	Gene expression (qRT-PCR, RNA seq) Western Blot Motion analysis Patch clamp Sharp electrode electrophysiology Ca^+2^ imaging Mitocondrial stress test- seahorse ELISA-cAMP level Nuclear magnetic resonance spectroscopy
[[21], [79]] Pointon et al., 2015; 2017	hiPSC-CM Primary human cardiac microvascular EC Primary human cFBs	Drug Screening	Confocal imaging-immunofluorescence	Gene expression (qRT-PCR) Motion analysis Cell viability assay (cellular ATP content)
[77] Ravenscroft et al., 2016	hESC-CM Primary human EC Primary human cFBs or FBs	Drug Screening Toxicity Testing	Confocal imaging-immunofluorescence	Gene expression (qRT-PCR) Motion analysis Ca^+2^ Imaging
[20] Richards et al., 2020	hiPSC-CM HUVECs Primary human cFBs	Disease modelling -myocardial infraction	Confocal imaging- immunofluorescence	Gene expression (qRT-PCR) Motion analysis Ca^+2^ imaging Mitochondrial stress test - seahorse
[30] Silva et al., 2020	hiPSC-CM	Cardiac development Cardiac maturation	Optical and light sheet microscopy – immunofluorescence Histology	Ca^+2^ imaging Patch clamp Gene expression (RNAseq)
[57] Lee et al., 2019	hESC-CM hESC-mesenchymal stem cells	Disease modelling Cardiac Fibrosis	Optical Microscopy Confocal imaging-immunofluorescence	Gene expression (qRT-PCR, microarray) FACS Motion analysis MEA analysis
[29] Hofbauer et al., 2021	hiPSC-CM hESC-CM Human cardiac microvascular EC Chicken embryo	Cardiac development	Optical Microscopy Confocal imaging-immunofluorescence Histology Electron microscopy	Ca^+2^ and voltage imaging Motion analysis Gene expression (RNAseq) Reporter cell lines Proteomics analysis

hPSC-CM immaturity has, however, been a challenge to modelling disease other than channelopathies and improving maturation state has been studied extensively over the last several years. Several reports have shown that culturing hPSC-CMs in 3D, mimicking the environment of cardiac tissue *in vivo*, improves the physiological and functional properties of CMs compared to 2D monolayers [[14], [15], [16]]. Moreover, if these 3D models also capture the complexity of native heart tissue, such as the communication between CMs and cardiac stromal- and vascular cells, then maturation is improved even further [[17], [18], [19], [20]]. Initially, primary endothelial cells (EC) such as those from human umbilical vein (HUVECs), or dermal fibroblasts (FB) were used in these 3D models but more recently it has been possible to generate ECs (specifically cardiac ECs) and cardiac FBs (cFB) from hPSC [18,21,22]. These have been combined in different ratios with CMs in microphysiological heart tissues (MHTs) (Table 1). The results broadly showed benefits for hPSC-CM maturation using cFB rather than dermal FBs and that this was mediated by junctional coupling with CMs [18]. 3D MHTs not only offer the advantage of being able to integrate multiple cell types but also microenvironmental cues, such as forced electrical or mechanical “pacing” or those mediated by the tissue scaffold, to closely mimic native heart. In this review, we first describe current MHT models, then summarize their applications with an overview of structural and functional readouts that can be used to report (patho-)physiology and drug/compound responses. Finally, we discuss how the field might move forward in refining and implementing these in the near future.

## 2 Current microphysiological tissue models

MHTs can be broadly divided into three types: those based on self-organized aggregates of cells (also referred to as spheroids, microtissues or organoids), those relying on biomaterials and bioengineering (engineered heart tissues (EHT)) and finally those in which perfusion can be integrated (Heart-on-chip). Human MHT models together with their cell sources, applications and readouts are summarized in Table 1, Table 2, Table 3. These tables are based on the selection from many studies in the field with a focus on more recent studies.

**Table 2:** Literature summary of EHTs.

Study	Cell types	Application	Readouts	
			Structural	Functional
EHTs				
[128] Dostanić et al., 2020	hiPSC-CM hiPSC-cFBs	Miniaturized cardiac tissues - Collagen/Matrigel hydrogel		Motion analysis Force measurement
[50,67,68,88] Nunes et al., 2013; Wang et al., 2019; Zhao et al., 2019; Feric et al., 2019	hiPSC-CM Human ventricular cFBs	Cardiac maturation Disease modelling Drug screening - Collagen/Matrigel/Fibrin hydrogel	Histology Confocal imaging- immunofluorescence Electron microscopy Second harmonic generation	Force measurement Motion analysis Ca^2+^ imaging Pharmacological analysis Gene expression (qRT-PCR; RNAseq) Electrophysiological measurements Western Blot Viability assay Pharmacological analysis
[38,126] Huebsch et al., 2015; 2016	hiPSC-CM Stromal cells	Miniaturized cardiac tissues - geometric confinment	Confocal imaging- immunofluorescence	Force measurement Motion analysis MEA analysis Ca^2+^ Imaging Patch-clamp Pharmacological analysis
[40] Leonard et al., 2018	hiPSC-CM	Cardiac Maturation - Fibrin hydrogel	Optical and confocal imaging- immunofluorescence Electron microscopy	Force measurement Motion analysis Gene expression (qRT-PCR) Ca2+ Imaging Western Blot
[89] Lind et al., 2017	hiPS-CM HUVEC Neonatal Rat Ventricular CM	Drug screening	Confocal imaging- immunofluorescence	Motion analysis ​+ ​sensors
[87] Lu et al., 2017	hiPSC-CM	Drug screening - Fibrin hydrogel	Confocal imaging- immunofluorescence Electron microscopy	Cell viability assay (MTS) Gene expression (qRT-PCR) Motion analysis Pharmacological analysis
[33,34] Ma et al., 2014; 2015	hESC-CM hiPSC-CM	Geometric confinement Disease modeling	Confocal imaging- immunofluorescence	Motion analysis MEA analysis Gene expression (qRT-PCR) Cell proliferation assay (EdU) Cell migration imaging Pharmacological analysis
[70] Macqueen et al., 2018	Neonatal rat ventricular CM hiPSC-CM	Disease modelling - Polycaprolactone (PCL)/gelatin nanofibres scaffold	Confocal imaging- immunofluorescence	Ca^2+^ Imaging Pressure-volume measurements Echocardiology
[85,86] Mannhardt et al., 2016; 2020	hiPSC-CM	Drug screening - Fibrin hydrogel	Confocal imaging- immunofluorescence Histology Electron microscopy	Motion analysis Pharmacological analysis Gene expression (RNAseq)
[66] Prondzynski et al., 2019	Human primary CM hiPSC-CM	Disease modelling - Fibrin/Matrigel hydrogel	Confocal imaging- immunofluorescence	Gene expression (RNAseq, nanoString nCounter) Western Blot Motion analysis Action potential measurement (electrodes and patch clamp)
[41] Ronaldson-Bouchard et al., 2018	hiPSC-CM human FBs	Cardiac maturation - Fibrin/hydrogel	Confocal imaging- immunofluorescence Electron microscopy	Force measurement Path-clamp Motion analysis Ca^2+^ imaging Gene expression (qRT-PCR) Pharmacological analysis Mitochondrial respirometry - Seahorse
[78] Takeda et al., 2018	hiPSC-CM	Drug cytotoxicity	Confocal imaging- immunofluorescence Histology	Gene expression (qRT-PCR) MEA analysis Cell viability assay (cellular ATP content) LDH assay Ca^2+^ imaging Motion analysis
[55] Tiburcy et al., 2017	hPSC-CM human FBs	Cardiac maturation Disease modelling - Collagen hydrogel	Cell composition analysis – Flow cytometry; Confocal imaging- immunofluorescence	Force measurement Motion analysis Gene espression (qRT-PCR, RNAseq) Action potential measurement – electrodes
[19,53] Mills et al., 2017, 2019	hESC-CM Neonatal Rat Ventricular CM	Cardiac Maturation Drug screening - Collagen/Matrigel Hydrogel	Optical and Confocal imaging - Immunofluorescence Cell composition analysis – Flow cytometry Electron microscopy	Motion analysis Ca^2+^ imaging Patch-clamp Gene expression (qRT-PCR, RNAseq) Mass Spectrometry - proteomic analysis; Central carbon metabolite analysis Mitochondrial respirometry - Seahorse
[63] Bailey et al., 2021	hiPSC-CM hiPSC-fibroblasts Primary macrophages	Disease modelling - SARS-CoV-2	Confocal imaging- immunofluorescence Histology Electron microscopy	Gene expression (qRT-PCR; RNAseq; RNAscope) Motion analysis Cell viability assay (TUNEL)

**Table 3:** Literature summary of HoCs.

Study	Cell types	Applications	Readouts	
			Structural	Functional
HoCs				
[83] Lee at al., 2021	HER-2-overexpressing human breast cancer cell line hiPSC – CM	Disease modeling	Optical microscopy – brightfield Confocal microscopy – immunofluorescence	Biosensors on micro-electrodes
[121] Maoz et al., 2017	hiPSC – CM	Drug screening	Electron microscopy Confocal microscopy – immunofluorescence	MEA analysis
[45] Marsano et al., 2016	neonatal rat ventricular CM hiPSC – CM	Disease modelling	Optical microscopy – brightfield Confocal microscopy – immunofluorescence	Motion analysis
[95] Mastikhina et al., 2020	human cFBs hiPSC – CM	Disease modelling	Optical microscopy – brightfield Confocal microscopy – immunofluorescence	Atomic force microscopy Gene expression (RNAseq) Pharmacological analysis
[46] Mathur et al., 2015	hiPSC – CM	Drug cytotoxicity	Optical microscopy – brightfield Confocal microscopy – immunofluorescence	Motion analysis Ca^2+^ imaging Pharmacological analysis
[47] Schneider et al., 2019	hiPSC – CM		Optical microscopy – brightfield Confocal microscopy – immunofluorescence	Motion analysis
[154] Schneider et al., 2022	hiPSC – CM human primary dermal fibroblasts		Optical microscopy – brightfield Confocal microscopy – immunofluorescence	Motion analysis Ca^2+^ imaging O_2_ sensors
[69] Wang et al., 2014	hiPSC -CM	Disease modelling	Confocal microscopy – immunofluorescence	Motion analysis
[82] Skardal et al., 2017	human hepatic stellate cells hiPSC – CM human lung microvasculature ECs human airway stromal mesenchymal cells human bronchial epithelial cells	Drug screening	Optical microscopy - brightfield Confocal microscopy - immunofluorescence	LC-MS metabolic profiling electromechanical biosensors
[153] Tanumihardja et al., 2021	hiPSC – CM		Electron microscopy	pH and O_2_ sensors
[48] Xiao et al., 2014	primary neonatal rat CM hESC-CM	Drug screening	Optical microscopy - brightfield Confocal microscopy - Immunofluorescence Electron microscopy	Motion analysis
[[125], [155]] Zhang et al., 2017; 2021	human primary hepatocytes hiPSC – CM	Drug screening	Brightfield microscopy - cellular morphology Fluorescence microscopy – cardiac markers	Electromechanical biomarkers sensors Motion analysis
[140] Shen et al., 2017	Murine ESC-CM hESC-CM	Cardiac maturation	Confocal microscopy – immunofluorescence	Ca^2+^ imaging RAMAN spectroscopy

Microtissues (or spheroids) and organoids commonly refer to 3D tissue models although these terms are not always used as originally described. Lancaster and Knoblich defined organoids as “self-organized tissues that develop from PSCs or progenitor cells by recapitulation of embryonic development” [23]. The term “spheroid” has traditionally been used to describe aggregates of terminally differentiated cells. Subsequently though, many groups used the word “organoid” to describe any collection of PSCs or adult SCs that were differentiated into region specific cells of the organ with the help of growth factors (reviewed in Ref. [24]). For the heart, all three terms have been used as well as “cardioids” to describe beating aggregates of differentiated hPSCs whether they have formed spontaneously or have been created by mixing multiple pre-differentiated heart cell types. These beating aggregates can be formed entirely scaffold-free, e. g. on a non-adhesive surface (see reviews [[25], [26], [27]] where cFBs produce their own extracellular matrix (ECM), or by integration of natural ECM or substitute scaffolds (e.g. hydrogels). These models generally have different applications and some are useful for studying early heart development [[28], [29], [30]] but it is of note that unlike for many organs, organoids cannot be formed from stem cells in the adult heart since despite earlier claims, it does not seem that such a population is present to any extent after birth [31]. This may in part explain why the adult mammalian heart repairs poorly after damage.

Integration of biomaterials and tissue engineering principles provides environmental cues that further benefit tissue organization, cell-cell communication and results in elasticity properties close to the human heart [[32], [33], [34], [35]]. One of the first EHTs was generated by seeding a mixture of cells and extracellular matrix around Velcro-coated glass posts which allowed contraction of tissues against resistance. These structures resembled *in vivo* cardiac muscle fibers and allowed direct measurement of contraction force from deflection of the posts [36]. Several groups adopted and improved this simple yet effective system to quantify the change in contractile force of tissues, for example upon external metabolic or electromechanical stimulus (reviewed in Ref. [37]).

Two main strategies were employed to generate the EHTs - geometric confinement and mechanical load. Providing appropriate geometric confinement to the tissue is important for tissue morphology and functionality. For example, a rectangular frame induced CMs to exhibit a more rod-shape morphology, uniaxial calcium flow and improved sarcomeric organization compared to square frames or monolayer cultures in multi-well plates [6,38]. In addition, physiological mechanical stretching is important for tissue development and maturation [[39], [40], [41]].

Microtissues and EHTs can recapitulate several physiological features of heart muscle tissue (multiple cell types, typical contraction rates, 3D structure). However, they lack perfusable vessel-like structures, which *in vivo* transport oxygen, nutrients and cells. Organ-on-chip (OoC) technologies offer opportunities to integrate perfusion through microfluidics and study neovasculogenesis of the tissue constructs; vessel structures and transport processes are thus created much like those *in vivo* (reviewed in Refs. [[42], [43], [44]]).

Heart-on-chip systems (HoCs) are typically devices made of polymer modules featuring microscale networks of channels and chambers. The chambers commonly house the microtissues, while the channels act as ducts that supply the tissues with nutrients, growth factors, gases, soluble compounds and (blood or immune) cells. The tissue chambers can be separated by a semi-permeable barrier (e.g. micro-channels or porous membranes) from the direct flow of fluid to protect the tissues from sheer stress, yet allow perfusion of nutrients [[45], [46], [47], [48]]. Some HoCs allow detection of contractile forces by introducing cantilevers that moved with CM contraction [49]. They integrate cyclic mechanic stimulation mimicking contraction of the heart which in turn induces the pulsating flow [45] or electrical pacing [50]. Depending on the targeted research question and experimental requirements, chip designs might differ from one another to suit the specific purpose but ultimately tailored or in-house designs create challenges for standardization. There are a number of systems now commercially available that address this to some extent by ensuring that at least the manufacturing process is standardized.

Collectively, MHT models are poised to advance the cardiovascular field as their potential to study CM maturation and disease models and carry out drug screening begins to be exploited.

## 3 Applications

Aside from supporting CM maturation, MHTs are already being implemented in several areas including some mentioned above: studying cardiogenesis, investigating disease mechanisms, identifying which cardiac cell types are responsible for pathophysiology, assessing cardiotoxicity and identifying potential drug therapies. For all applications, models from any of the three MHT categories might be most suitable depending on the specific research questions. The model of choice should be as simple as possible but as complex as necessary.

### 3.1 Mediating cardiomyocyte maturation

One application of MHT models is to study intercellular communication in the heart which is important for heart development and maturation. Integration of CMs with ECs into microtissues improved structure and functionality of hPSC-CM [51]. Inclusion of cFBs enhanced CM maturation in their contraction force and frequency, gene expression, Ca^2+^ handling, sarcomere assembly and β-adrenergic receptor signaling proteins [17,18]. In addition, 3D tissue formats prevented cFBs forming stress fibers compared to monolayer cultures and eliminated profibrotic growth factors for cFB activation and *trans*-differentiation into myofibroblasts [17]. Furthermore, combining all three cell types (CMs, ECs and cFBs) in these tissues increased intracellular cAMP levels in CMs through CM-cFB crosstalk; this was further enhanced by Endothelin-mediated signaling from ECs to CMs [18]. Together, this resulted in better assembly of Connexin43 (Cx43) gap junctions and further promoted maturation of CMs. This was in line with *in vivo* studies [52]. Crosstalk between CMs and FBs and resulting maturation was not seen when dermal FBs replaced cFBs [18]. Dermal FBs express low or negligible levels of Cx43, depending on the particular batch which might explain why they are less effective in mediating maturation compared to cFBs. However, other tissue sources of FBs may express Cx43 and promote maturation. In addition, any paracrine effects of FBs may be independent of Cx43.

In addition to co-culture, other approaches to tissue maturation have used EHTs, in which systematic optimization of ECM components and regulation of exogenous mechanical- and metabolic factors (including energy substrates) have resulted in significant benefit [41,[53], [54], [55]].

These studies demonstrated that multiple cues must be carefully orchestrated to govern distinct aspects of the adult CM phenotype. Therefore, although much progress has been made and there are multiple ways to induce a postnatal CM phenotype, none is yet definitive and obtaining fully mature/adult CMs in MHTs (or any other model) remains a challenge.

### 3.2 Disease modelling

2D hPSC-CMs are easy to generate and use and are adequate for modeling ion channelopathies and cardiomyopathies caused by specific mutations, as mentioned earlier [51]. However, for more complex disorders, MHTs provide better structural, functional and metabolic maturation as well as allowing integration of multiple cell types and simulation of environmental conditions. These 3D models have also been used for studying inflammation subsequent to myocardial infarction or other injury [20,56]; or fibrosis [57,58].

Bacterial or viral infection can affect the heart. Severe acute respiratory syndrome coronavirus (SARS-CoV-2) infection causes coronavirus disease (COVID-19). Many groups worldwide are investigating viral infection and disease mechanisms using human stem cell models. Although the predominant pathology is observed in the respiratory system, several studies have shown that the cardiovascular system is also affected by this virus [59,60]. 20–30% of all COVID-19 patients suffer from severe cardiac symptoms and increased levels of cardiac protein troponin I in their blood which indicates heart damage and is linked to increased risk of morbidity [61,62]. 3D culture systems discussed here are also increasingly used to investigate how inflammation causes heart damage and the effects of therapy on the heart [[63], [64], [65]].

Taking advantage of the ability to integrate differentiated cardiac cells from healthy- (genetically corrected) and patient derived hiPSCs, multiple cardiac pathologies such as hypertrophy [[66], [67], [68]], arrhythmogenic cardiomyopathy [18], long QT syndrome type 3 (LQT3) [33] and Barth syndrome [69] have been modelled using different MHTs. Aside from providing insights into disease mechanisms, they have also been used to identify potential therapeutics [69,70].

These models are potentially useful for providing insights into disease mechanism but to date they have lacked immune/inflammatory cells, involved in triggering or sustaining these pathological processes [71], or nerve cells that can invade heart excessively and cause arrhythmias after myocardial infarction [72].

### 3.3 Cardiotoxicity assessment

Cardiotoxicity is still a major reason that drugs in development fail to reach the clinic or are labelled as high risk after market entry. Rodents do not always accurately predict drug effects on humans because the physiological parameters like contraction kinetics such as the beat rate of the human heart differ significantly from those in widely used laboratory animals (reviewed in Ref. [73]). In addition, direct translation of drug efficacy and toxicology from rodents to humans is limited due to differences in pharmacokinetic properties. Large animals such as non-human primates or dogs are more predictive but have financial and ethical burdens. Alternative models to animals are thus of growing interest.

One of the first major milestones towards using hiPSC-CMs among these alternatives is the Comprehensive *in vitro* Proarrhythmia Assay (CiPA) initiative, a collaboration between academia, pharmaceutical industries, regulatory authorities and stem cell companies [74]. This showed a good correlation between the assay outcome in hiPSC-CMs and known toxicity in humans. In a later study, simultaneous measurement of contraction kinetics, action potentials and Ca^2+^ transients using voltage and calcium-sensitive dyes, was shown to be even more predictive of cardiac toxicity [75,76]. It is an ongoing effort to assess the drug-induced proarrhythmic risk using hiPSC-CMs and emerging *in vitro/in silico* assays. Collectively, these studies provided evidence that assays based on hiPSC-CMs could eventually replace traditional toxicology studies with the combination of 3D *in vitro* models and *in silico* tools.

MHT platforms are also being used to test functional toxicity of a number of drugs [21,77,78]. In these studies, tissues exhibited alterations in cardiac contraction in response to inotropic or non-inotropic drugs with an ability to predict *in vivo* outcome with high sensitivity and specificity [21]. Moreover, these models were able to distinguish between negative and positive inotropic compounds as opposed to previous studies on hiPSC-CM monolayer cultures [79]. This confirmed improved accuracy of risk assessment for a spectrum of compounds. Structural cardiotoxicity of FDA approved compounds was also assessed [80]. Morphological assessment of drug-exposed MHTs showed changes in structural integrity and cellular viability. Together, these studies revealed the potential value of MHTs in drug screening even though, because of their size and formats, they are not yet suitable for large scale compound screens. Pharmaceutical companies are increasingly adopting MHTs and more specifically OoC platforms for drug effectivity assessment and disease modelling [81] with current efforts on more standardized protocols.

Addressing drug metabolization and multi-organ effects, especially in OoC platforms integrating multiple organ systems with perfusion, are providing new opportunities to evaluate possible side effects of drug treatment and off-target tissues. For example, a heart-liver-lung multi-organ on chip platform showed that bleomycin, an anti-cancer drug that causes lung inflammation, also induced cardiotoxicity [82]. Another example was described by Lee at al. (2021), who connected HoC to a breast-cancer-on-chip to examine chemotherapy induced cardiotoxicity [83]. Combining cancer patient-derived hiPSC-CMs in this model could in the future aid prediction of individual cardiotoxic risk.

### 3.4 Development of cardiac therapeutics

Complementary studies also used hiPSC-CMs to predict drug-induced electrophysiological- and contractile changes (reviewed in Ref. [84]). 2D hiPSC-CMs and 3D EHTs [85,86] were compared for their (blinded) response to a list of known drugs. This resulted in highest predictivity and sensitivity from 3D-EHTs based on measuring force of contraction although combined measurement of contraction, action potential and calcium transients in 2D hiPSC-CM monolayer culture also gave high predictivity as mentioned above [76,84].

Several EHT and HoC drug screening platforms are scalable and have shown drug responses comparable to *in vivo* clinical observations [19,[87], [88], [89]]. Scalability (and corresponding cost) is important for pharma in large compound screens for therapeutics. EHTs and HoCs have additional advantages for some drug screens in providing an endothelial (or vascular wall) barrier (in Transwell® inserts). Drugs can be added on the basal side of the endothelial barrier whilst the cardiac tissue is on the apical side [89]. These models allow assessment of drug availability through the vasculature. Together, these platforms are increasingly used to develop *de novo* cardiac therapeutics.

## 4 Readouts

How cell responses can best be measured *in situ* preferably in real-time, is a crucial question for their utility. Readouts constitute an integral part of any model and are necessary to validate MHTs prior to implementation. Two important features of these heart models are relevant to translation: cardiac structure and function. In this section, we summarize the commonly used assays, both *in situ* in real time and terminal; these are summarized in Table 1, Table 2, Table 3

### 4.1 Microscopy-based analysis of MHT assembly and composition

CMs in the heart are tightly coupled to create the structure necessary for highly synchronized, repetitive contraction and electrical conduction. This is evidenced as aligned sarcomeres that form characteristic “bands” in the heart. Thus, proper physiology requires defined 3D structure in the tissue. MHTs may thus include architectural guidelines to shape the *in vitro* model e.g. scaffolds, spatial constraints or anchoring points. However, the structure of cardiac microtissues can vary from spheroid-like to extended fibers. Analysis of the tissue structure is thus highly relevant to demonstrate enhanced maturation or quantify pathological changes in disease. Here, we summarize microscopic methods and their readouts used for this purpose.

When designing MHTs, the material properties, such as transparency, refractive index and organic solvent compatibility, must be considered. In addition, the thickness of the material should be taken into account for imaging with short or long-distance objectives. If the platform does not have the same footprint as microscope slides or plates, tailored holders are needed that fit on the microscope stage. Moreover, when live imaging is preferred, an on-stage incubator for climate control is required.

The main ways to visualize MHT structure and its cellular components are label free imaging, immunofluorescence and use of fluorescent reporter cell lines. Label-free [89] and reporter cells [90] do not require additional steps such as antibody staining before imaging. On the other hand, immunofluorescent labelling may have higher specificity for protein detection and allow multiple proteins to be imaged simultaneously. Another post-fixation labelling technique is standard histology where various cellular and intracellular structures are visible and can be characterized [50]. In addition to labelling, tissue clearing techniques are increasingly used to improve image quality. 3D tissue imaging might be limited due to the scattered light by the lipids, intracellular or extracellular fluids present in the tissue. Tissue clearance eliminates these components and allows penetrate of light into the tissue by keeping refractive indices equal [91,92]. As a result, thicker tissue samples can be visualized by different microscopic techniques while preserving the 3D integrity of the sample.

Optical microscopy is the gold standard to image tissue structures. It includes a wide range of techniques from widefield to high-resolution microscopy. Transmitted light microscopy, such as bright-field or phase contrast, provides information on microtissue compaction, shape and motion (see Table 1, Table 2, Table 3) without requiring additional labeling or fixation, and is suitable for live imaging. However, transmitted light microscopy is usually unable to distinguish specific cell types and their intracellular structures. Confocal microscopy and super-resolution microscopy overcome this. They also provide high spatial resolution with the possibility of optical sectioning, imaging in the Z-direction and 3D-reconstruction although this adds additional labor and time in the post-imaging process [93]. However, photo damage remains a limitation of these techniques. In addition, higher magnification objectives and corresponding short working distances (<1 ​mm) may require removing the microtissue from the platform [94] which may be challenging and affect structural integrity of the microtissue. Two-photon microscopy can also provide 3D information. In comparison to confocal microscopy, it has better penetration depth due to greater wavelengths of photons used for imaging allowing visualization of deeper regions. It does not require optical clearance of the tissue and reduces photobleaching. Additionally, second harmonic generation allows label-free imaging of specific cell structures, like collagen deposition [67,95]. Light sheet microscopy based on illuminating an entire plane of tissue by laser in the form of a light sheet, has proven particularly useful for imaging 3D objects in particular because photo-bleaching is lower than conventional confocal imaging. Although not yet broadly applied to MHTs, first reports indicate advantages for tissue imaging on microfluidic chips [96].

Electron microscopy uses electron beams instead of light to generate a projection image. Compared to optical microscopy, electron microscopy provides much higher resolution. However, it requires fixed samples and often tissue slices, which make it a terminal assay. It also presents tissue collection challenges for HoC platforms [38,97].

### 4.2 Functional readouts

Functional analysis of cardiac tissue models can broadly be divided into four approaches: molecular (e.g. protein or gene expression), electrophysiological, mechanical and metabolic readouts.

#### 4.2.1 Molecular analysis

Molecular analysis is not generally regarded as a direct functional readout but it does reveal expression profiles and pathways relevant for functionality. Commonly used assays for both *in vivo* and *in vitro* studies is high-throughput next generation sequencing (NGS) for transcriptome, genome and epigenome analysis. Besides well-known techniques like RT-PCR, RNA sequencing (RNAseq) is used increasingly either on bulk cell populations, where it reports average signal expression in cell populations, or on single cells. Bulk measurements on thousands to millions of cells is straightforward but does not distinguish different cell populations thereby potentially missing rare events that could be highly relevant to a disease mechanism [[98], [99], [100]]. By contrast, single cell analysis allows tracking of individual cell populations and detection of rare events (reviewed in Refs. [[101], [102], [103], [104], [105]]). RNAseq has been used for microtissues, organoids and EHTs but not so far for HoCs, probably due to difficulties in extracting the tissues from chips. Whilst highly informative, RNAseq lacks the corresponding protein expression profiles and require disruption of tissues and cells. Disruption itself can upregulate stress genes [106] and thereby introduce artefacts and spatial information is lost. Batch-to-batch and line-to-line variability may in the future be addressed by automation of procedures. Alternative high content imaging methods e. g Co-detection by indexing (CODEX) [107], imaging mass cytometry [108], MACSima imaging cycling staining (MICS) [109] or RNAscope [110] complement the toolbox for protein and RNA expression profiling. Spatial transcriptomics, although not yet commonly applied to MHTs offer next generation methodology for *in situ* tissue profiling [111]. Other approaches include collecting tissue-conditioned media for biomarker detection or recent impedimetric immunosensors with antibody coated electrodes which transmit electrical signals when biomarkers or pathogens bind to them [112]. These methods are currently used for quality control of embryos derived from *in vitro* fertilization where invasive cell sampling carries high risk [[113], [114], [115], [116]]. Clinical cardiac biomarkers [117,118] can also be used on conditioned media and all of these non-invasive methods allow long-term monitoring of live tissues.

#### 4.2.2 Electrophysiology

Electrophysiological properties such as the action potential (AP) profile of the heart reflect functionality of CMs. Rapid ion transfer in and out of membrane channels alters the membrane voltage which results in AP changes. The AP starts with depolarization of the membrane by sodium ion influx and continues with initial repolarization by potassium (K^+^) ion efflux and a plateau phase in which calcium ions (Ca^+2^) move inwards. Further repolarization by K^+^ currents bring the membrane potential back to the resting level of −85 mV. Intracellular recordings of electrophysiological parameters are obtained using sharp electrodes or single cell patch clamp; these remain the benchmark for quantification of functionality in microtissues and EHTs. Voltage sensitive dyes [119] and integration of (non-invasive) electrodes are alternatives for extracellular recordings of field potential. Voltage sensitive dyes can be used in any platform and is suitable for whole tissue analysis [120]. However, they can be cytotoxic and are sensitive to photobleaching. Multielectrode arrays (MEAs) are culture plates with embedded electrodes [121] which are widely used for real-time estimation of QT-prolongation (an important safety parameter) [122] and conduction velocity [123]. They are mostly used for 2D cell cultures as it is a challenge to record the whole tissue electrophysiology considering their limited surface interface between the electrodes and the tissue. Adopting a similar strategy, self-rolling biosensor arrays which wraps around the 3D microtissue/organoid [124], or microelectrodes integrated in EHT and HoC platforms allow continuous and multiplexed recording of electrophysiological signals [88,121,125].

#### 4.2.3 Mechanics

Excitation-contraction coupling is one of the key indications of a functional cardiac tissue and Ca^2+^ is one of the key ions of this process. Sarcoplasmic reticulum releases Ca^2+^ ions in response to electrical excitation during the AP plateau phase which increases the level of intracellular Ca^2+^. Coupling and uncoupling of intracellular Ca^2+^ with troponin C in myofilaments activates and deactivates contraction, respectively. In order to reflect the physiological properties of the 3D models with respect to their Ca^2+^ level changes, fluorescent dyes with a variety of excitation ranges and Ca^2+^ affinities are used. Although this technique is real-time, fast and efficient, it is not suitable for long-term analysis and sensitivity of recordings depends highly on the type of the dye. Genetically modified hPSC lines that encode a calcium indicator (GECI) circumvents limitations of Ca^2+^ dyes and offer user-independent possibilities to measure and analyze Ca^2+^ flux and contractility [126].

Optical tracking of cells, tissues, beads [127], pillars or cantilevers around which tissues have compacted [88,128] is another way to retrieve contraction parameters as relative pixel displacement or absolute contraction force. Traction force microscopy, fractional shortening of sarcomere length and micropost displacement are examples of such techniques mainly used for single cells [54,129]. Software such as MuscleMotion [130] and OpenHeartWare [47] are user-friendly and open access tools for optical tracking of live contracting tissue from video recordings or image stacks. This type of automated contraction analysis enables quantification of contraction changes in diseased and healthy states or in response to drugs and can also be used for MHTs discussed earlier (Table 1, Table 2, Table 3 [47,131]). However, precise and consistent tracking becomes a challenge with increased heterogeneity in contrast and texture of 2D or 3D models; this may create measurement artefacts. In addition, preload dependent mechanics cannot be well analyzed with optical tracking. Therefore, classical isometric force measurements remain the gold standard in biomechanical characterizations of the heart muscle. Integrated sensors in MHTs allow continuous electronic examination of contractile stress and beat rate [89]. Such analysis does not require invasive methodology and is user-friendly.

#### 4.2.4 Metabolic readouts

The adult heart has a low energy reserve. To meet the high energy demand, CMs produce ATP from several metabolic substrates (e.g. glucose, pyruvate, triglycerides, glycogen, lactate, ketone bodies, fatty acids (FAs) and some amino acids) based on environmental conditions [132,133]. During early heart development, glucose and lactate are the major energy sources and glycolysis the major metabolic pathway to produce ATP. Postnatally though, the mitochondrial oxidative capacity increases and FA beta oxidation becomes the major energy source [134,135]. Recapitulating this “metabolic switch” from glycolysis to mitochondrial oxidative phosphorylation is an important reflection of maturity in MHTs [53].

##### 4.2.4.1 Oxygen consumption and glycolysis measurements

Several commercially available kits and instruments (e.g. BioProfile Analyzers and Biochemistry Analyzers) allow quantification of extracellular glucose and lactate levels by colorimetric and fluorometric detection in cell culture media and are thus non-invasive. The Seahorse (extracellular flux) XF Analyzer is a fast and comprehensive approach that allows simultaneous recording of the oxygen consumption rate (OCR) and extracellular acidification rate (ECAR) of live cells and tissues. Three main metabolic assays can be performed with this technology: (I) mitochondrial stress; (II) glycolytic stress and (III) palmitate oxidation [136].

##### 4.2.4.2 Quantification of metabolites and cell secretome

Conventional biochemical assays (RNAseq, LC-MS, ELISA) are widely used to detect metabolic changes in static cultures such as EHTs and microtissues/organoids and in a time-resolved manner for perfused systems. Secretome analysis can provide insight into disease mechanisms [137] and microenvironmental changes in the ECM, for example during hPSC differentiation into CMs [138]. However, proteome and secretome data acquisition and interpretation are challenging, even when applied to simple systems (e.g. monocultures). Low concentration of analytes, serum components in the media, protein splice variants can all result in false negatives and positives; this might be the reason why this technique has not been widely adopted in more complex cardiac systems (e.g. co-culture of different cell types).

Nuclear magnetic resonance spectroscopy (NMR) detects extra-/intra-cellular metabolites in MHTs in tissue supernatants and lysates. The technique can be used non-invasively and the data gives valuable input in the metabolic state of the tissues [18]. Alternatively, RAMAN spectroscopy provides non-invasive measurement of intracellular metabolites, and it is already in use to monitor hPSC-CM maturity [139,140] as well as ECM components in tissues [141,142].

## 5 Perspectives

MHTs have become much more robust and reproducible over the last several years but individually, each system still has issues to overcome before widespread application in the cardiovascular field. MHTs based on hPSCs can mimic aspects of embryonic development and integrate multiple (pre-differentiated) cell types. Cell types that have not yet been integrated into these models include (i) cardiac resident macrophages (CRM) that link with CMs and can regulate electrical activity and cardiac homeostasis [[143], [144], [145]] and (ii) the cardiac autonomic nervous system that mediates the electrical and mechanical signals from the brain to the heart [146]. Additionally, the intrinsic cardiac nervous system (ICNS) with its sensory, motor and interconnecting neurons regulates electrical conduction in the heart [147]. Several studies have indicated important roles of CRMs [145,148,149] and ICNS (reviewed in Ref. [150]) in cardiac pathophysiology *in vivo.* Moreover, many differentiation protocols, cell lines and platform designs mean little standardization between the systems, precluding the direct comparison of outcomes. One example of the challenge this presents is that identical EHTs made from different hiPSC lines exhibited different baseline contractile force, kinetics, and beating rate [86]. It is unclear whether this derives from different genetic backgrounds, line-specific disease associated SNPs or gene variants or just different state-of-maturity of the CMs used in the EHTs. In addition, efforts towards more head-to-head comparisons including batch-to-batch and clone-to-clone variability are needed.

Self-organized microtissues and organoids miss the full complement of micro-environmental cues that direct physiological maturation of CMs. EHTs can introduce natural or synthetic microenvironments that together with mechanical cues go some way to increasing physiological realism. Likewise, HoCs with microchannels have been developed which can integrate perfusion and fluidic flow, the critical feature of native tissues. In addition, most platforms are fabricated by academic labs which produce devices in small series and only few are at present commercially available. In addition, physical and chemical properties of materials may be variable with respect to absorption or retention of small molecules in the culture medium which could lead to misinterpretation of results. It is important to characterize these properties prior to use.

MHTs nevertheless offer remarkable opportunities in many applications including disease modelling and drug screening in the cardiac field particularly if populated with human cells. Data produced using current validation assays suggests that these systems can recapitulate many complex physiologies of heart tissue in healthy and diseased states with respect to structure, functionality and metabolism. However, most of these assays have not yet been used in HoCs, therefore lack options to mimic (blood and lymph) vascular flow. Most HoC designs are closed so tissues are not easily accessible for readouts. For this reason, terminal (or end-point) readouts are often preferred. Moving towards non-invasive assays and adapting these to be compatible with HoCs will be one way of addressing and providing alternatives for this issue. Integration of biosensors in chip design is an option. However, most commercial systems, for example, do not allow in-house integration of these sensors, perhaps explaining why many labs prefer their own in-house designs.

For structural characterization, high resolution imaging of 3D tissues along the Z-axis is still challenging in HoCs since most are still too thick to be imaged using conventional confocal microscopy. This means that the tissue must be removed from the platform, making high resolution imaging an end-point readout. Even though not often reported for HoCs, light-sheet microscopy and 2-photon microscopy can offer alternatives for direct on-chip imaging of live tissues and combination with transgenic cell lines expressing fluorescent marker proteins provide non-invasive structural readouts.

Metabolic studies are mainly focused on O_2_ consumption and glycolysis. However, most techniques are not suitable for real-time measurement in HoCs as they need either tissue lysis or effluent collection over several hours of perfusion. For example, although the Seahorse-XF is widely used on cell monolayers, microtissues and EHTs (Table 1, Table 2), most of the microfluidic devices are made of PDMS which is highly permeable for oxygen. Hence oxygen consumed by the tissues is replenished almost instantly downstream. In addition, they are mostly closed systems where the cell or tissue proximity is not easily accessible by instrumentation and the culture medium is delivered through continuous flow. The design flexibility however, allows, in principle for the integration of the sensors directly into the chip platform. Alternatively, non-invasive fluorescent-lifetime imaging microscopy (FLIM) allows characterization of mitochondrial metabolic oxidative changes by means of endogenous fluorescence in live samples [151].

Quantification techniques for metabolites and secretome (ELISA, MS, NMR and others reviewed in Ref. [133]) are used for microtissues and EHTs, they have not yet been adapted for HoC systems. However, some of these techniques have been applied in other OoCs, for example Raman spectroscopy in pancreas-on-chip [152].

Integrated or modular sensors offer *in situ* real-time automated tools for metabolic readouts. Integrated sensors include electrochemical sensors to measure oxygen and pH analytes as a difference of electrical current or potential [153]. However, they can also be adapted to measure non-electrically active species such as glucose and lactate. These sensors often require a transducer to detect signals. On the other hand, optical sensors can replace transducers and detect signals from a simple transparent window on top of the sensor. Integrated sensors can be positioned close to the tissue allowing real-time measurement [154]. However, they are in the system for entire culture periods which in some cases last multiple weeks while the measurement is only relevant when the system is challenged, for example with drugs. This means for enzyme-based sensors (but also others) that they might already be degraded before they are used. Integrated sensors are single use which significantly increases the cost per chip.

Modular multisensors, on the other hand, can be re-usable and thus cost efficient. They may contain several physical sensors for monitoring pH, oxygen and temperature or electrochemical sensors [155]. Modular multisensor systems in combination with modular OoC platforms can be useful to detect differential secretion of enzymes in different organs, for example in response to different compounds [82]. However, they are dependent on the flow rate and some measurements, such as oxygen consumption in PDMS chips can be impaired if not positioned properly [155]. Electrochemical and optical sensor alternatives and their integration in OoC systems have recently been extensively reviewed [156].

Emerging technologies like FLIM, Raman spectroscopy and integrated sensors may greatly contribute to non-invasive metabolic analysis of living tissues inside HoC platforms.

## 6 Conclusions

In sum, we reviewed here different 3D cardiac tissue generation platforms in combination with hiPSC-derived cardiac cell types together with their applications and readouts. These models are emerging tools to investigate cardiac development and pathophysiology. Further refinement in the generation and readout methods might eliminate hurdles that have been discussed here and allow closer mimicry of the adult heart with a possibility to standardize, reduce costs and adopt less labor-intensive methodology.

## Declaration of competing interest

J.N and D.E. are employees of Miltenyi Biotec B.V. & Co. KG.
